# Study on the Polishing Mechanism of Composite Magnetic Field-Controlled Internal Flow Channels in Additive Manufacturing

**DOI:** 10.3390/ma19112390

**Published:** 2026-06-03

**Authors:** Hao Li, Rui Wang, Jinxu Zhang, Suhuan Guo, Guosheng Su, Jin Du, Binxun Li, Peirong Zhang, Yan Xia, Yujing Sun

**Affiliations:** 1 Shandong Key Laboratory of CNC Machine Tool Functional Components, School of Mechanical Engineering, Qilu University of Technology (Shandong Academy of Sciences), Jinan 250353, China; lihao258099@163.com (H.L.); zjx1323083@163.com (J.Z.); sgs@qlu.edu.cn (G.S.); dj84105@126.com (J.D.); libx@qlu.edu.cn (B.L.); zhpr@qlu.edu.cn (P.Z.); yxia@qlu.edu.cn (Y.X.); 2 Shandong Institute of Mechanical Design and Research, Jinan 250031, China; 3 Zhejiang University-University of Illinois Urbana-Champaign Institute, International Campus, Zhejiang University, Haining 314400, China; rui.23@intl.zju.edu.cn; 4 Shandong Key Laboratory of Surface Engineering and Intelligent Equipment for Key Metal Components, School of Mechanical Engineering, University of Jinan, Jinan 250022, China; 15726390103@163.com

**Keywords:** additive manufacturing, internal flow channels, magnetic abrasive finishing, material removal model, surface quality improvement

## Abstract

Surface defects in additively manufactured internal channels limit their practical applications. Conventional post-processing methods suffer from limited accessibility and a tendency toward over-polishing, whereas magnetic abrasive finishing (MAF) offers high adaptability and precise process controllability. This study systematically investigates the material removal mechanisms in magnetic abrasive polishing and clarifies the distinctions and transitions between two-body and three-body wear modes. Based on these findings, a rolling removal model grounded in rough surface contact theory and a sliding removal model incorporating correction factors are established. Experiments were conducted on AlSi10Mg internal channels fabricated via selective laser melting (SLM) using a composite magnetic field polishing apparatus. The results verify the accuracy of the proposed models and demonstrate that the process effectively reduces surface defects and surface roughness. Although some deviations arise from model idealization and non-uniform magnetic field distribution, this study establishes a systematic theoretical framework for material removal in additively manufactured complex internal channels.

## 1. Introduction

Additive manufacturing (AM), also known as 3D printing, fabricates complex, lightweight components layer by layer directly from digital models, facilitating efficient production without requiring molds [1]. AM is extensively applied in aerospace (e.g., components with internal flow channels), biomedicine (e.g., porous implants), defense, and sports equipment [2,3,4,5,6]. However, AM is susceptible to defects, including spheroidization, porosity, step effects, interlayer lack of fusion, and thermal stress cracking [7]. These defects lead to rough surfaces, dimensional deviations, and stress concentrations, which can severely compromise fatigue life, fluid performance, and sealing integrity [8]. To mitigate the process defects associated with additive manufacturing, various post-processing methods—such as electrochemical or chemical polishing using chemical reagents, hydraulic-driven abrasive flow polishing, and composite polishing that combines multiple techniques—have been developed and can generally satisfy standard processing requirements [9]. Nevertheless, these methods remain insufficient for intricate internal geometries, where issues related to tool accessibility, surface uniformity, and chemical contamination persist. Consequently, there is a critical need for novel or optimized post-processing strategies capable of addressing these challenges, particularly in components with complex internal features.

In contrast to conventional post-processing techniques, magnetic abrasive finishing (MAF) offers distinct advantages. It is capable of processing multi-phase alloys containing non-conductive phases and is environmentally friendly, as it generates no chemical waste. By regulating the forces acting on abrasive particles through a magnetic field, MAF prevents abrasive embedding, over-polishing, and clogging of internal flow channels. The process is stable and highly controllable, thereby ensuring the integrity and uniformity of complex internal surfaces [10]. The working principle involves the formation of a flexible magnetic brush from abrasive particles under a magnetic field, enabling it to conform to complex surfaces for full-area polishing. For non-magnetic workpieces, an external magnetic field can be applied to generate a magnetic brush within internal cavities. Furthermore, the MAF process exhibits high controllability, strong adaptability to workpiece geometry, and self-sharpening abrasive particles that continuously renew to extend service life [11]. Magnetic abrasives typically consist of ferromagnetic and abrasive phases [12], and their performance can be further enhanced through material and process optimization. For example, Zhang et al. [13] introduced Mn as a sintering additive.

The Archard wear model [14,15], originally proposed by Archard, is a classical theoretical framework for quantitatively predicting material removal. It was initially developed to analyze adhesive wear mechanisms. Building on this model, Rabinowicz [16] modified the removal formulation using a grain shape coefficient, assuming that all grains participate in cutting and that the plowed material is entirely converted into wear debris. This assumption renders the model overly idealized. Garrison and Garriga [17] further refined Rabinowicz’s equation by incorporating elastic–plastic deformation and work hardening effects. In addition, Zum Gahr [18,19] introduced a material removal ratio factor based on single-particle scratching experiments. However, existing material removal models for magnetic abrasive polishing remain limited. At the mechanistic level, these models often reduce the complex removal process to a single mechanism, whereas actual material removal arises from the interaction and evolution of multiple mechanisms. Furthermore, the empirical coefficients lack universality, which limits the accurate prediction of material removal behavior in materials with complex microstructures. Therefore, achieving precise prediction of the polishing process for complex workpieces requires the development of optimized models that integrate multiple removal mechanisms.

In our previous study published in the Journal of Manufacturing Processes (2025) [20], a composite magnetic field polishing apparatus was developed and the macroscopic polishing performance of SLM AlSi10Mg internal channels was experimentally evaluated. The present work differs fundamentally from that study by focusing on the mechanistic interpretation and mathematical modeling of localized abrasive-particle interactions. Specifically, this work establishes a rolling-sliding transition framework to explain the coupling relationship between abrasive motion modes and material removal mechanisms under composite magnetic field conditions.

Researchers have conducted a comprehensive review of magnetic grinding of internal surfaces in modern metal additive manufacturing parts [21,22,23], pointing out a severe lack of well-defined dynamic material removal models capable of handling complex topographies. By regulating the magnitude of the normal magnetic field force and defining the motion patterns using torque balance equations, the study enables the application of corresponding removal models to surfaces with varying roughness. This study elucidates the material removal mechanisms in magnetic abrasive polishing and establishes corresponding removal models. It clarifies the differences and interrelationships between the microscopic mechanisms of two-body and three-body wear. The results indicate that the mode of abrasive particle motion is the key determinant of the removal mechanism and provide a theoretical framework for subsequent differentiated modeling. Separate removal models are developed for the two dominant motion modes, namely rolling and sliding. The rolling model is based on the Greenwood–Williamson contact theory and describes material removal induced by the accumulation of plastic strain during repeated rolling of abrasive grains. The sliding model is based on micro-cutting mechanisms and incorporates a correction factor for the plow groove ratio. These models provide a theoretical basis for subsequent experiments and predict the results of the polishing experiments.

## 2. Theoretical Removal Model

### 2.1. Abrasive Removal Mechanism

The abrasive particle removal mechanism is generally classified into two types based on particle involvement: two-body wear and three-body wear. Two-body wear occurs when abrasive particles are fixed or embedded on the tool surface and move relative to the workpiece via rolling or sliding, resembling a micro-cutting process that generates grooves or scratches. In contrast, three-body wear occurs when free abrasive particles act as an intermediate medium between two surfaces and remove material through repeated rolling and sliding actions. These two mechanisms fundamentally govern both the microscopic behavior and macroscopic efficiency of material removal.

**Figure 1:**
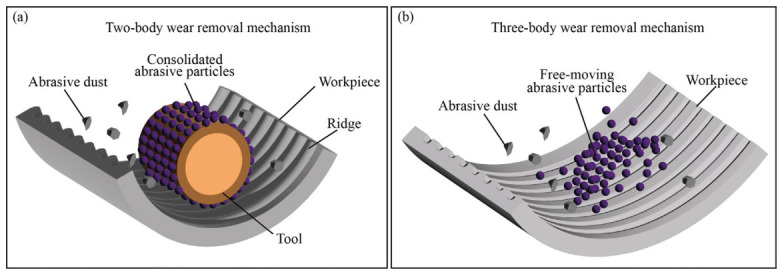
Schematic representations of two-body and three-body wear mechanisms. (**a**) Two-body wear mechanism; (**b**) three-body wear mechanism.

Material is continuously removed via the sliding or rolling of abrasive particles in two-body wear. In contrast, three-body wear involves a combination of rolling and sliding motions of free abrasive particles. Rolling leads to discontinuous material removal. However, changes in process parameters or surface topography may cause particles to transition from free rolling to temporary embedding and sliding, resulting in behavior similar to two-body wear. Therefore, two-body and three-body wear can transform into each other under certain conditions. This dynamic transition makes it difficult to clearly distinguish removal modes at the microscopic scale and complicates the development of corresponding theoretical models, as shown in Figure 1.

Since material removal models correspond to different abrasive particle motion and stress states, they must be capable of identifying particle motion states and selecting the corresponding removal mechanisms. This requires that the model is not restricted to a single mechanism but can switch according to critical transitions in particle motion, thereby evolving from single deterministic models to composite removal models. Therefore, a deep understanding of two-body and three-body wear and their transition conditions is essential for constructing accurate and practical polishing prediction models.

At the fundamental level of wear mechanisms, there exists a direct correspondence between abrasive particle motion patterns and material removal mechanisms. In three-body wear, sliding-dominated abrasive particles exhibit pronounced tangential relative motion with the workpiece surface. The sustained tangential force induces direct shear removal of material under normal loading, resulting in a continuous and efficient ploughing wear surface. In contrast, rolling-dominated abrasive particles rely on repeated normal loading to fracture the surface layer, leading to material removal via cumulative plastic deformation. Because the material removed per cycle is limited, multiple loading cycles are required, resulting in a significantly lower material removal rate compared with sliding wear.

### 2.2. Contact Method of Abrasive Particles

In magnetic abrasive polishing, abrasive particles are subjected to complex magnetic-mechanical interactions that directly govern the material removal behavior. Under the action of the composite magnetic field, abrasive particles experience magnetic forces, normal support forces from the workpiece surface, and tangential frictional forces generated at the contact interface. In addition, adjacent abrasive particles may interact through magnetic chain coupling forces. Abrasive particles are also theoretically influenced by gravitational force and centrifugal force induced by rotational motion.

Considering the microscale dimensions and relatively low inertial effects of the abrasive particles, the influences of gravitational force and centrifugal force are assumed to be negligible. Furthermore, due to the highly dynamic and stochastic nature of interparticle magnetic interactions, quantitative characterization of particle-chain coupling behavior remains extremely difficult. Therefore, to maintain analytical tractability and establish a simplified mechanistic framework, the present study analyzes the polishing behavior at the level of an individual abrasive particle.

Accordingly, the following assumptions are adopted in the theoretical modeling process:

(1) Abrasive particles are simplified as rigid spherical bodies;
(2) Gravitational force, centrifugal force, and interparticle magnetic interactions are neglected due to their comparatively small influence on localized contact behavior;
(3) Material removal is assumed to originate from localized abrasive-surface interaction under magnetic loading conditions.

The interaction between normal loading, tangential friction, and particle motion state governs the transition between rolling-dominated and sliding-dominated removal mechanisms. Establishing the force interaction relationship at the microscopic contact interface therefore provides the basis for mechanistic modeling of material removal behavior, prediction of polishing performance, and interpretation of surface morphology evolution.

**Figure 2:**
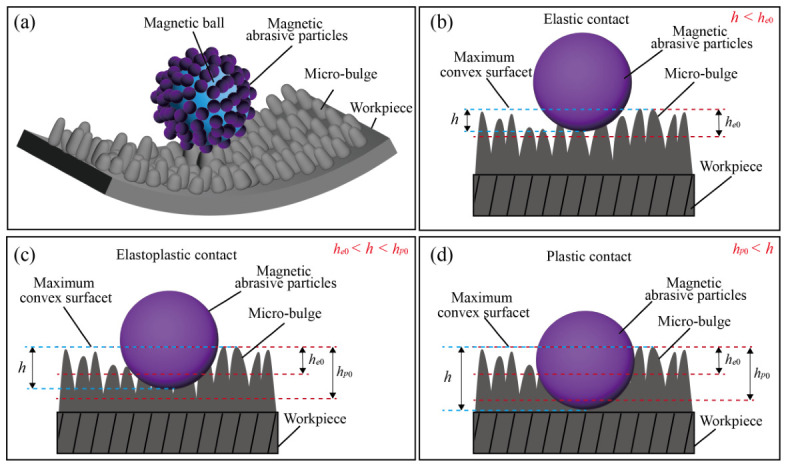
Abrasive contact modes. (**a**) Surface contact of abrasive particles; (**b**) Elastic contact; (**c**) Elastoplastic contact; (**d**) Plastic contact.

Within the framework of contact mechanics, Johnson’s contact theory [24] classifies the contact states between abrasive grains and the workpiece surface into three regimes according to the normal load and indentation depth (Figure 2). During the elastic contact regime, the normal load remains low and the contact stress stays below the material yield limit. Only reversible elastic deformation occurs, and no material is removed. Under elastic–plastic contact conditions, the normal load exceeds the yield limit and induces plastic deformation in the contact zone. Partial elastic recovery occurs upon unloading. Both micro-cutting and plastic deformation contribute to material removal. When the contact reaches the fully plastic regime, plastic deformation dominates the entire contact zone and results in permanent material removal.

The understanding and control of contact state transitions are of critical importance in magnetic abrasive polishing. By regulating key parameters, such as magnetic field strength and abrasive particle properties, the interaction between abrasive particles and the workpiece can be maintained within a desired contact regime, enabling controlled and efficient material removal and improved surface integrity.

Therefore, applying the Johnson contact mechanics model to identify and predict contact modes is a key theoretical step toward precise process control.

Based on Johnson’s theory, the following approach can be derived:

When *h* < *h_e_*_0_, the contact is elastic. Within this range, the expression for the normal force *F_t_* is:

(1) $$F_{t}=\frac{4}{3}R^{\frac{1}{2}}E^{*}h^{\frac{3}{2}}$$

where *R* is the radius of the abrasive particle, E* is the composite elastic modulus, *h* is the penetration depth.

When *h_e_*_0_ < *h* < *h_p_*_0_, the contact is elastic. Within this range, the expression for the normal force *F_t_* is:

(2) $$F_{t}=\frac{4}{3}\pi R\sigma_{y}h\left\{2+\ln\left[\frac{1}{4}\frac{E^{*}}{\sigma_{y}}{\left(\frac{2h}{R}\right)}^{\frac{1}{2}}+\frac{1}{3}\right]\right\}$$

where *R* is the radius of the abrasive particle, *σ_y_* is the yield strength of the workpiece, *h* is the penetration depth, E* is the composite elastic modulus.

When *h_p_*_0_ < *h*, the contact is elastic. Within this range, the expression for the normal force *F_t_* is:

(3) $$F_{t}=2.9\pi R\sigma_{y}h$$

where *R* is the radius of the abrasive particle, *σ_y_* is the yield strength of the workpiece, *h* is the penetration depth.

Where

(4) $$h_{e0}=0.64\pi^{2}\frac{R\sigma_{y}^{2}}{E^{{*}^{2}}}\;h_{p0}={\left(\frac{9\pi }{4}\right)}^{2}{\left(\frac{\sigma_{y}}{E^{*}}\right)}^{2}R$$

*h_e_*_0_ is the indentation depth of the abrasive grain when the contact stress at the contact surface reaches the elastic limit, *h_p_*_0_ is the indentation depth of the abrasive particle when full plastic deformation occurs, *R* is the radius of the abrasive particle, *σ_y_* is the yield strength of the workpiece, E* is the composite elastic modulus.

(5) $$\frac{1}{E^{*}}=\frac{1-\nu_{k}^{2}}{E_{k}}+\frac{1-\nu_{i}^{2}}{E_{i}}$$

*E_k_* and *E_i_* are the elastic moduli of the workpiece and abrasive, *ν_k_* and *ν_i_* are the Poisson’s ratios of the workpiece and abrasive.

### 2.3. The Motion of Abrasive Particles

Under three-body wear conditions, the workpiece surface exhibits point-like or short-arc indentations, randomly oriented plough marks, or their combination under varying normal loads. These features arise from the stochastic motion of abrasive particles, making the identification of their motion patterns essential for establishing a material removal model. Abrasive particles exhibit multiple motion modes, including sliding, rolling, spinning, and their combinations. Among these modes, sliding and rolling dominate the material removal process. Sliding-induced removal is analogous to micro-cutting in two-body wear, whereas rolling-induced removal results primarily from cumulative plastic deformation under repeated compression. Rotational motion is transient and unstable, and its contribution to material removal is negligible. Combined sliding–rolling motion is also unstable in practice due to the significantly lower rolling friction compared with sliding friction. According to the principle of minimum energy dissipation, abrasive particles preferentially adopt the rolling mode whenever possible. Therefore, theoretical models of three-body wear typically consider only sliding and rolling as the dominant motion modes. Analyzing the transition conditions between these modes is essential for predicting surface topography and controlling material removal behavior. To this end, a quasi-static mechanical model of magnetic abrasive particles under magnetic field and workpiece constraints is required.

Unlike conventionally manufactured aluminum alloys, SLM-fabricated AlSi10Mg exhibits a highly heterogeneous microstructure resulting from rapid solidification during the laser powder bed fusion process. The material consists of a supersaturated α-Al matrix surrounded by eutectic Si networks, accompanied by melt pool boundaries, partially melted particles, and staircase surface defects. Consequently, the local mechanical properties, including hardness and yield behavior, are spatially non-uniform across the internal channel surface. During magnetic abrasive finishing, abrasive particles may interact differently with distinct microstructural regions. These localized variations may influence the rolling-sliding transition behavior of abrasive particles and contribute to the stochastic nature of material removal during polishing. To maintain analytical tractability, homogenized material properties were adopted in the present model.

**Figure 3:**
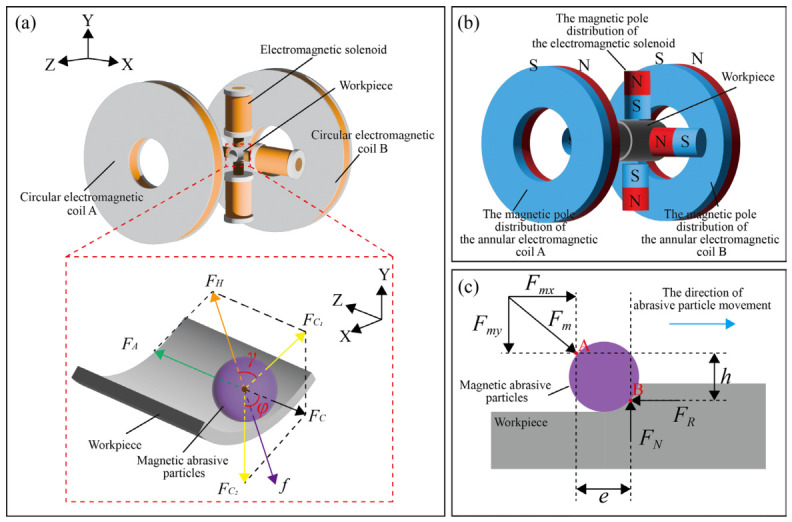
Force analysis of abrasive particle motion. (**a**) Magnetic abrasive force; (**b**) magnetic pole distribution; (**c**) abrasive force analysis.

A single spherical abrasive particle is primarily subjected to a composite magnetic force generated by the circumferential electromagnetic solenoid and the axial annular electromagnetic coil (Figure 3a,c). In addition, it experiences normal support and tangential friction from the workpiece surface. The composite magnetic force at point A is decomposed into tangential and normal components relative to the workpiece surface. At the contact point (point B), the workpiece exerts a normal support force and a tangential friction force on the abrasive particle.

Fang Liang et al. [25] proposed a torque-balance-based method to determine the motion state of spherical abrasive particles. This method is based on the critical equilibrium condition at the onset of motion, where the force balance satisfies *F_mx_* = *F_R_* and *F_my_* = *F_N_*. The determination of the motion mode depends on the magnitude of the torque about point A or point B. Taking point B as the pivot point, the driving torque for rolling is *F_mx_* · *h*, while the resisting torque is *F_my_* · *e*, where *h* and *e* denote the corresponding lever arms relative to point B.

The torque equilibrium criterion can be expressed as:

When *F_mx_* · *h* > *F_my_* · *e*, the driving torque is dominant, the abrasive particles roll.

When *F_mx_* · *h* ≤ *F_my_* · *e*, the driving torque is not enough to overcome the resistance torque, the abrasive particles slide.

The lever arms *e* and *h* are functions of particle size, material properties, and the applied normal force, and are given as follows:

(6) $$e=\frac{4}{3}\frac{\sqrt{\pi^{2}D_{0}^{4}H^{2}-F_{my}^{2}}}{\pi^{2}D_{0}H}$$

(7) $$h=\frac{2}{3}\frac{\left(\pi^{2}D_{0}^{4}H^{2}-10^{8}F_{my}^{2}\right)\sqrt{\pi^{2}D_{0}^{4}H^{2}-F_{my}^{2}}}{\pi^{3}D_{0}^{5}H^{3}}$$

where *D*_0_ is the abrasive particle diameter, *H* is the workpiece hardness.

Furthermore, by incorporating the actual magnetic field distribution of the experimental device (Figure 3b), Equations (6) and (7) and the corresponding decision criteria are further specified, leading to the formulation of Equations (8) and (9).

Rolling:

(8) $$F_{c_{2}}<F_{N}=\frac{D_{0}^{2}H\sqrt{\pi^{2}-2\pi \frac{\cos\gamma }{\tan\varphi }}}{10^{4}}$$

Sliding:

(9) $$F_{c_{2}}⩾F_{N}=\frac{D_{0}^{2}H\sqrt{\pi^{2}-2\pi \frac{\cos\gamma }{\tan\varphi }}}{10^{4}}$$

where *F_c_2__* is the normal component of magnetic force, *F_N_* is the critical normal force, *D*_0_ is the abrasive particle diameter, *H* is the workpiece hardness, *γ* is the angle between the combined external force and the tangential component of the magnetic force. *φ* is the angle between the magnetic direction and the normal component of the magnetic force.

### 2.4. Polishing Path of Cylindrical Inner Flow Channel

A common simplification in kinematic modeling of magnetic abrasive polishing treats abrasive particles as point masses, thereby neglecting the influence of particle volume and shape on their trajectories. Under the action of a composite spatial magnetic field, the particles move in practical equipment. The tangential magnetic force generated by the circumferential electromagnetic solenoid drives the particles to move along a circular path on the inner wall at a constant angular velocity. Meanwhile, the axial annular electromagnetic coil is alternately energized, generating a periodic oscillating magnetic field along the workpiece axis, which induces axial reciprocating motion of the abrasive particles. The actual motion of magnetic abrasive particles is a superposition of these two fundamental motions, which is used to describe their trajectory within the cylindrical inner flow channel.

**Figure 4:**
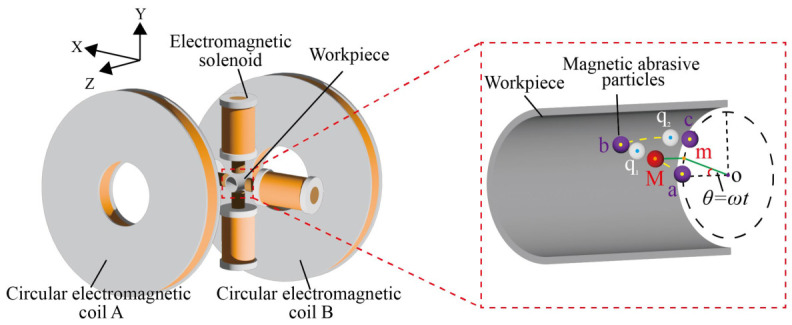
Schematic illustration of the experimental setup and corresponding abrasive particle displacement behavior.

As shown in Figure 4, within the X–Y plane (cross-section of the inner flow channel), abrasive particles undergo uniform circular motion driven by a rotating magnetic field. The time variable is denoted by *t*. The spatial position of an abrasive particle is represented by M (*x*, *y*, *z*), and its projection onto the XOY plane is given by m (*x*, *y*, 0). Accordingly, the particle motion in the X–Y plane can be described by the following kinematic equation:

(10) $$\left\{\begin{array}{l} x=r\cos\omega t\\ y=r\sin\omega t \end{array}\right.$$

where *ω* is the angular acceleration of the abrasive particle, *r* is the inner radius of the cylindrical inner flow channel.

Along the Z-direction, particles exhibit an approximately uniformly accelerated reciprocating motion induced by an alternating axial magnetic field, with a constant friction coefficient *μ* assumed. Starting from point a, the particle moves toward point q_1_ with uniform acceleration *a*_1_ induced by the magnetic field generated by coil A. At point q_1_, coil A is deactivated and coil B is activated, resulting in uniform deceleration under the combined effects of the opposing magnetic force and friction until the velocity reduces to zero at point b. Subsequently, with periodic switching of the magnetic field, the particle reverses its motion and returns to point a, completing one full motion cycle. The spatial displacement of the abrasive particle over one cycle is obtained as the superposition of multiple motion segments. Accordingly, the particle trajectory can be expressed as:

(11) $$\left\{\begin{array}{c} x=r\cos\omega t_{1}\\ y=r\sin\omega t_{1}\\ z_{1}=\frac{1}{2}a_{1}t_{1}^{2} \end{array}+\left\{\begin{array}{c} x=r\cos\omega t_{2}\\ y=r\sin\omega t_{2}\\ z_{2}=-\frac{1}{2}a_{2}t_{2}^{2}+\upsilon_{1}t_{2} \end{array}+\left\{\begin{array}{c} x=r\cos\omega t_{3}\\ y=r\sin\omega t_{3}\\ z_{3}=-\frac{1}{2}a_{3}t_{3}^{2} \end{array}+\left\{\begin{array}{c} x=r\cos\omega t_{4}\\ y=r\sin\omega t_{4}\\ z_{4}=\frac{1}{2}a_{4}t_{4}^{2}-\upsilon_{3}t_{4} \end{array}+\cdots \right.\right.\right.\right.$$

In the formula, *t*_1_ is the time from point a to point q_1_, *z*_1_ is the displacement from point a to point q_1_, *a*_1_ is the acceleration from point a to point q_1_, *t*_2_ is the time from point q_1_ to point b, *z*_2_ is the displacement from point q_1_ to point b, *a*_2_ is the acceleration from point q_1_ to point b, *υ*_1_ is velocity of the abrasive particle at point q_1_, *t*_3_ is the time from point b to point q_2_, *z*_3_ is the displacement from point b to point q_2_, *a*_3_ is the acceleration from point b to point q_2_, *t*_4_ is the time from point q_2_ to point c, *z*_4_ is the displacement from point q_2_ to point c, *a*_4_ is the acceleration from point q_2_ to point c, *υ*_3_ is velocity of the abrasive particle at point q_2_.

Under the assumption that the switching times of the axial magnetic fields are negligible (i.e., *t*_2_ and *t*_4_), and that the magnetic forces generated by the two coils are symmetrically distributed, the axial motion of abrasive particles can be approximated as a periodic repetition of uniformly accelerated segments between points a and b.

Accordingly, using Equations (10) and (11), the total path length of the abrasive particle during machining is obtained by integrating the arc length of its motion over the total processing time, as follows:

(12) $$L=\frac{t}{t_{AB}}\int_{0}^{t_{1}}\sqrt{{\left(\omega r\right)}^{2}+{\left(a_{1}t\right)}^{2}}dt$$

where *t* is the total processing time, *t_AB_* is the time interval between the action of the annular electromagnetic coil A and B, *t*_1_ is the displacement time from point a to point b, *ω* is the angular acceleration of the abrasive particle, *r* is the inner radius of the cylindrical inner flow channel, *a*_1_ is the linear acceleration of the abrasive particle.

### 2.5. Single Abrasive Particle Rolling Removal Model

To describe the probabilistic interaction between rolling abrasive particles and the rough additively manufactured surface, a simplified statistical asperity-contact framework inspired by the Greenwood–Williamson (GW) [26] theory was employed. It should be noted that the original GW model was derived for elastic asperity deformation based on Hertzian contact assumptions. In the present study, the framework is utilized in a simplified manner to characterize the statistical distribution of localized abrasive-surface contacts under rolling-dominated polishing conditions.

**Figure 5:**
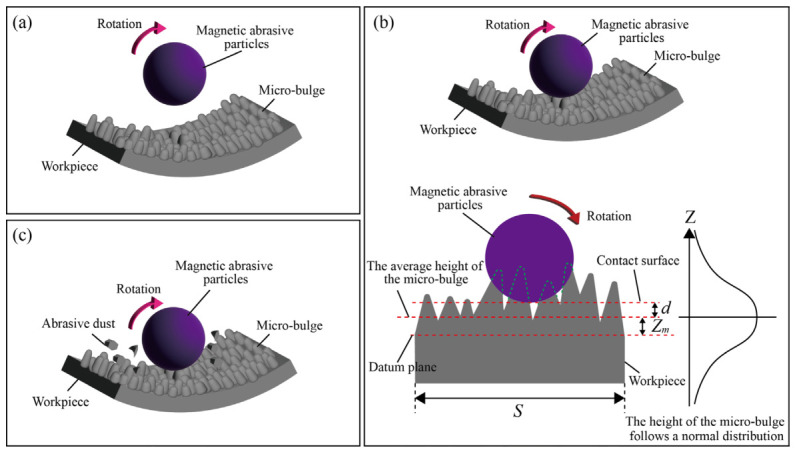
Schematic of the material removal mechanism based on the rolling model. (**a**) Before contact; (**b**) Material removal begins; (**c**) Material removal completed.

During actual material removal, rolling and sliding mechanisms often occur simultaneously and interact with each other. The rolling mechanism is governed by plastic deformation induced by repeated compression, whereas the sliding mechanism is dominated by micro-cutting. For modeling simplification, the rolling removal model is constructed under the assumption that material removal is primarily governed by plastic deformation, while the contribution of micro-cutting is neglected. The base of each spherical abrasive particle is modeled as a smooth rigid plane, whereas the rough workpiece surface is represented as an assembly of spherical micro-asperities with randomly distributed heights and identical radii of curvature (Figure 5b).

The rolling removal model is formulated as follows:

(1) Probability of contact and number of contact points

Let *d* denote the separation distance between the spherical abrasive particle and the mean plane of the surface asperity height distribution. Asperities with height *z* > *d* are assumed to be in contact with the abrasive particle, resulting in plastic deformation and material removal.

Assuming that the asperity heights follow a Gaussian distribution, the probability density function is expressed as:

(13) $$\Phi \left(z\right)=\frac{1}{\sqrt{2\pi }\sigma }e^{-{\left(\frac{z}{2\sigma }\right)}^{2}}$$

The probability of contact for a single asperity is expressed as:

(14) $$p\left(z>d\right)=\int_{d}^{\infty }\Phi \left(z\right)dz$$

*N*′ denotes the total number of surface asperities. The number of asperities in contact can be expressed as:

(15) $$n=N\prime \int_{d}^{\infty }\Phi \left(z\right)dz$$

(2) Contact area of a single asperity and total contact area

Under fully plastic deformation conditions, when an individual asperity comes into contact with an abrasive particle, its contact area *A_i_* is governed by the deformation height *ω* (where *ω* = *z* − *d*) and the asperity radius of curvature *β*, and can be expressed as:

(16) $$A_{i}=\lambda \pi \beta \omega$$

In the above expression, λ = 2 denotes the fully plastic deformation regime.

Accordingly, the total plastic deformation area generated by all contacting asperities can be expressed as:

(17) $$A=\sum A_{i}=N^{\prime }\lambda \pi \beta \int_{d}^{\infty }\left(z-d\right)\frac{1}{\sqrt{2\pi }\sigma }e^{-{\left(\frac{z}{2\sigma }\right)}^{2}}dz$$

(3) Incorporation of surface statistical parameters and abrasive particle geometry

The total number of surface asperities is given by *N*´ = *ηA*_0_, where *η* denotes the asperity density and *A*_0_ is the nominal contact area. For spherical abrasive particles, the contact region at a given indentation depth is approximated as a circular area with radius, *α* leading to *A*_0_ = *πα*^2^.

Taking the mean asperity height as the reference plane, the maximum asperity height is denoted as *M*. The total actual contact area is obtained by integrating the asperity height distribution, with the upper integration limit set to *M*.

(18) $$A=2\eta \pi^{2}\alpha^{2}\beta \int_{d}^{M}\left(z-d\right)\frac{1}{\sqrt{2\pi }\sigma }e^{-{\left(\frac{z}{2\sigma }\right)}^{2}}dz$$

In the above expression, *d* = *M − h*, where *h* represents the normal indentation depth of the abrasive particle.

(4) Calculation of material removal mass

The total material removal mass is determined by the number of active abrasive particles, the total plastic contact area, the sliding path length on the workpiece surface, and the material density of the workpiece. Accordingly, the total material removal mass in the rolling removal model can be expressed as:

(19) $$M_{a}=\rho \cdot A\cdot L\cdot N=2\rho L\eta \pi^{2}\alpha^{2}\beta N\int_{d}^{M}\left(z-d\right)\frac{1}{\sqrt{2\pi }\sigma }e^{-{\left(\frac{z}{2\sigma }\right)}^{2}}dz$$

where *ρ* is the mass density of the workpiece, *A* is the total plastic contact area, *L* is the path length of a single abrasive particle, *N* is the number of magnetic abrasive particles involved in the removal, *η* is the surface density of the asperity on the workpiece surface, *α* is the indentation radius of the contact area after the abrasive particles are pressed into the workpiece, *β* is the radius of the asperity, *M* is the maximum peak height of the asperity, *d* is the distance between the contact surface of spherical abrasive particles and the average height plane of asperity, *z* is the height coordinate of asperity based on the average height of asperity, *σ* is the standard deviation of the peak height distribution of the workpiece surface profile.

The number of magnetic abrasive particles removed is:

(20) $$N=\frac{m_{1}}{m_{2}}=\frac{m_{1}}{\left[\rho_{1}\left(\frac{a}{a+b}\right)+\rho_{2}\left(\frac{b}{a+b}\right)\right]\cdot \frac{4}{3}\pi R^{3}}$$

In the formula, *m*_1_ is the total abrasive mass, *m*_2_ is the single abrasive mass, *ρ*_1_ is the iron matrix mass density, *ρ*_2_ is the abrasive mass density, *a*:*b* is the proportion of iron matrix and abrasive, *R* is the abrasive radius.

The radius of the slight protrusion is:

(21) $$\beta =\frac{\Delta^{2}}{\left(-z_{i+1}+2z_{i}-z_{i-1}\right)}$$

In the formula, *z*_i_ > *z*_i−1_ and *z*_i_ > *z*_i+1_, Δ is the sampling interval.

The standard deviation of the peak height distribution of the workpiece surface profile is:

(22) $$\sigma =\sqrt{\frac{\sum_{i-1}^{n}{\left(z_{i}-\mu \right)}^{2}}{n}}$$

In the formula, *μ* is the overall mean, *n* is the total amount of data.

In experimental analysis, a sample is used to represent the population, and the sample standard deviation is defined as:

(23) $$S=\sqrt{\frac{\sum_{i=1}^{n}{\left(z_{i}-\bar{z}\right)}^{2}}{n-1}}$$

In the formula, $\bar{z}$ is the sample mean, *n* is the sample size.

This model establishes a statistical framework that connects stochastic microscale plastic deformation with macroscopic material removal behavior, enabling the prediction of rolling-dominated wear and material removal processes.

### 2.6. Single Abrasive Particle Sliding Removal Model

During sliding removal, material removal is primarily governed by micro-cutting mechanisms. Abrasive grains directly remove material in the form of chips, while a small amount of material is displaced to the groove sides due to the ductility of the workpiece and forms pile-up. For modeling simplification, the sliding removal model is formulated based on the dominant micro-cutting mechanism, and the contribution of plastic deformation is neglected.

**Figure 6:**
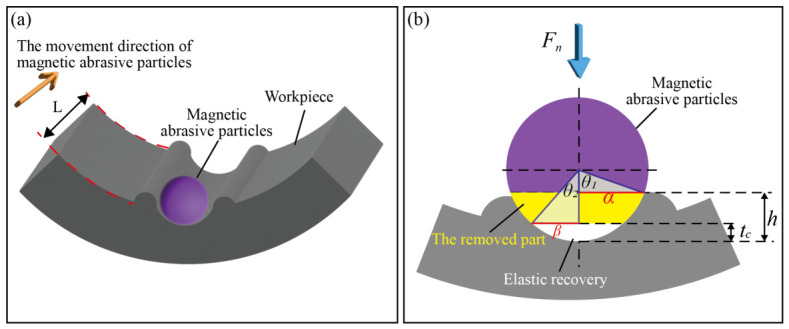
Schematic of the sliding-based material removal model. (**a**) Sliding removal mechanism; (**b**) cross-sectional geometry of abrasive particles.

According to Johnson’s contact theory, the abrasive particles and the workpiece are in an elastoplastic contact state under normal loading, and partial elastic recovery occurs during unloading. Accordingly, the actual material removal volume is determined by subtracting elastic recovery from the theoretical groove removal volume, as illustrated in Figure 6b.

(1) Geometric model and volume calculation

A spherical magnetic abrasive particle of radius *R* is subjected to a normal load *F*, leading to sliding micro-cutting during indentation into the workpiece. The cross-section of the theoretical groove is approximated as a circular segment, and the corresponding groove volume *V*_1_ is obtained as the product of the cross-sectional area and the sliding path length, and is given by:

(24) $$V_{1}=\left[R^{2}\cdot \mathrm{arc}\sin\left(\frac{\alpha }{R}\right)-\alpha \cdot \left(R-h\right)\right]\cdot L$$

where *R* is the radius of the spherical magnetic abrasive particles, *α* is the chord length related to the indentation geometry, *h* is the indentation depth of the abrasive particles, *L* is the path length of the abrasive particles.

Following polishing, the groove depth partially recovers due to elastic recovery of the material. The corresponding groove volume *V*_1_ after elastic recovery is defined as:

(25) $$V_{2}=\left[R^{2}\cdot \mathrm{arc}\sin\left(\frac{\beta }{R}\right)-\beta \cdot \left(R-t_{c}\right)\right]\cdot L$$

In the above expression, *R* is the radius of the spherical magnetic abrasive particles, *β* is the chord length after elastic recovery, *L* is the path length of the abrasive particles, *t_c_* denotes the total elastic deformation within the contact area, and is given by:

(26) $$t_{c}={\left(\frac{9}{16R}\right)}^{\frac{1}{3}}{\left(\frac{F_{n}}{E^{*}}\right)}^{\frac{2}{3}}$$

where *R* is the radius of the spherical magnetic abrasive particles, *F_n_* is the normal force, E* is the composite elastic modulus between the abrasive particles and the workpiece, the calculation formula is shown in Equation (5).

(2) Introduction of the furrow ratio coefficient

**Figure 7:**
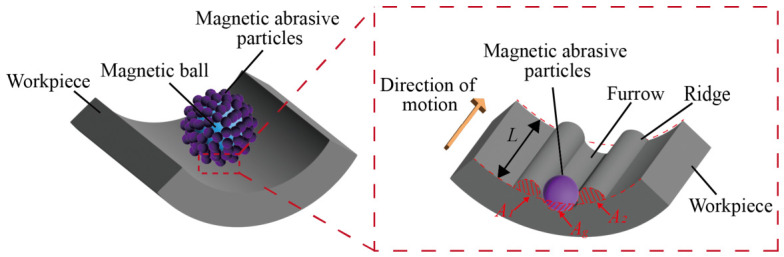
Furrow ratio coefficient.

Material removal during elastoplastic sliding cutting is inherently complex. As shown in Figure 7, even when elastic recovery is neglected, only a fraction of the removed material is converted into chips, while the remainder is extruded to the groove sides, forming permanent plastic pile-up ridges. Therefore, the actual chip volume is significantly smaller than the theoretical groove volume.

Accordingly, Zum Gahr [18,19] introduced a furrow ratio coefficient *f*_ab_ to correct the sliding removal model, defined as the fraction of material actually converted into chips, as given in Equation (27). Where 0 < *f*_ab_ < 1, its value is governed by material properties and abrasive particle geometry and must be determined experimentally.

(27) $$f_{ab}=\frac{A_{g}-\left(A_{1}+A_{2}\right)}{A_{g}}$$

In the equation, *A_g_* is the cross-sectional area of the furrow, *A*_1_ and *A*_2_ are the cross-sectional areas of the plastic ridges on both sides.

(3) Total material removal mass model

The total material removal mass in sliding mode can be expressed by accounting for elastic recovery, the furrow ratio coefficient, and the number of active abrasive particles:

(28) $$M_{b}=f_{ab}\cdot \rho \cdot N\cdot L\cdot \left\{R^{2}\cdot \left[\mathrm{arc}\sin\left(\frac{\alpha }{R}\right)-\mathrm{arc}\sin\left(\frac{\beta }{R}\right)\right]+R\left(\beta -\alpha \right)+\alpha h-\beta {\left(\frac{9}{16R}\right)}^{\frac{1}{3}}{\left(\frac{F_{n}}{E^{*}}\right)}^{\frac{2}{3}}\right\}$$

In the formula, *f*_ab_ is the furrow ratio coefficient, *ρ* is the density of the workpiece material, *N* is the number of magnetic abrasive particles involved in the removal, *L* is the path length of the abrasive particles, *R* is the radius of the spherical magnetic abrasive particles, *α* is the chord length related to the indentation geometry, *β* is the chord length after elastic recovery, *h* is the indentation depth of the abrasive particles, *F_n_* is the normal force, E* is the composite elastic modulus.

## 3. Experiment

### 3.1. Experiment Preparation

**Figure 8:**
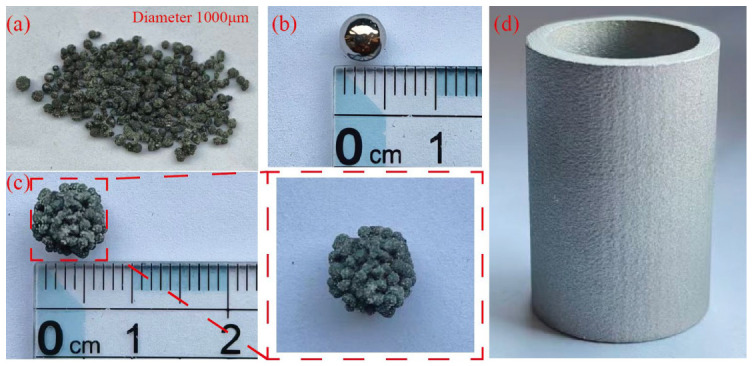
Preparation of polishing materials and specimens. (**a**) Magnetic abrasive particles; (**b**) Nd_2_Fe_14_B magnetic bead; (**c**) composite magnetic bead–abrasive particles; (**d**) internal flow channel specimen.

To verify the material removal rates predicted by different removal models, the experiments employed a spherical magnetic pole-based magnetic abrasive polishing system. In this configuration, magnetic abrasive particles (MAPs) were used to validate the rolling removal model, whereas magnetic bead–magnetic abrasive particles (MB–MAPs) were adopted for validation of the sliding removal model. The composite magnetic abrasive particles used in this study were developed by research group [13], and their morphology is shown in Figure 8a.

The workpieces used in this study are AlSi10Mg cylindrical internal flow channels fabricated by SLM, with dimensions of 30 mm in length, 16 mm in inner diameter, and 20 mm in outer diameter. The geometric parameters of the internal flow channel are summarized in Table 1.

**Figure 9:**
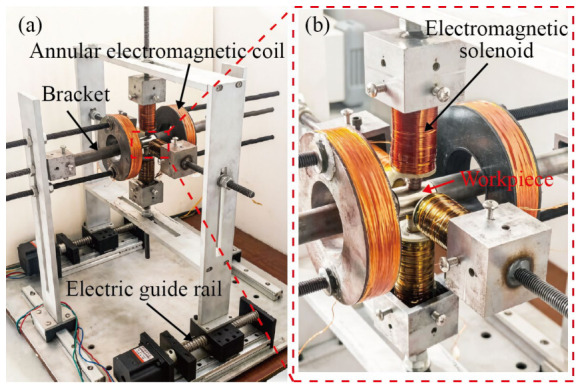
Composite magnetic-field magnetic polishing system. (**a**) Experimental setup; (**b**) polishing position.

To validate the proposed material removal model and realize controllable polishing, Guo et al. [20] developed a magnetically controlled polishing system based on a composite magnetic field, as shown in Figure 9. The system comprises four circumferentially distributed electromagnetic solenoids and two axially symmetric annular coils. Independent regulation of coil currents enables precise control of both the direction and magnitude of the magnetic field. The alternating gradient magnetic field is generated via periodic modulation of the circumferential solenoid currents, inducing rotational motion of the magnetic abrasive particles along the inner wall for circumferentially uniform processing. Meanwhile, the axial oscillating magnetic field generated by the annular coils produces reciprocating axial motion, ensuring complete coverage of the internal surface.

### 3.2. Inner Flow Channel Experiment of Magnetic Abrasive Polishing

During the development of the magnetic abrasive polishing model, the normal force serves as a governing parameter. According to Johnson’s contact mechanics theory, the relationship between the normal force acting between the magnetic bead and abrasive particles and the indentation depth is established, providing a theoretical basis for the single-particle sliding removal model. By integrating experimentally measured normal force data with torque balance analysis, the sliding removal model is formulated, and the furrow ratio coefficient is determined.

**Figure 10:**
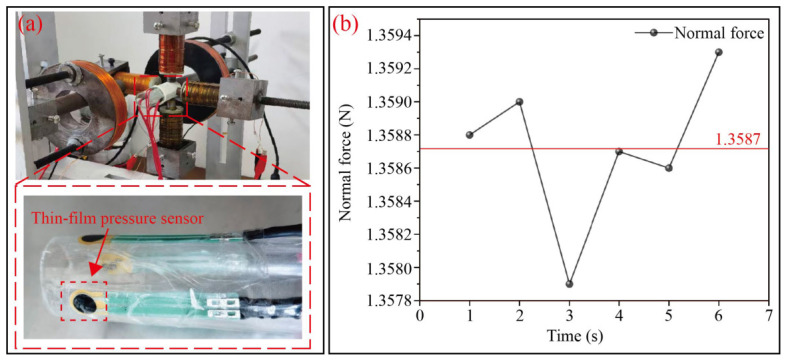
Magnetic bead–abrasive particles normal force measurement. (**a**) Force sensor installation; (**b**) Normal force magnitude.

A thin-film pressure sensor integrated with a magnetic bead–magnetic abrasive polishing tool was employed to measure the normal force experimentally, as shown in Figure 10b. The theoretical normal force calculated from the indentation depth is 1.471 N, whereas the average experimentally measured value is 1.359 N. The relative error is 8.26%, demonstrating good agreement between the theoretical prediction and experimental measurements.

**Figure 11:**
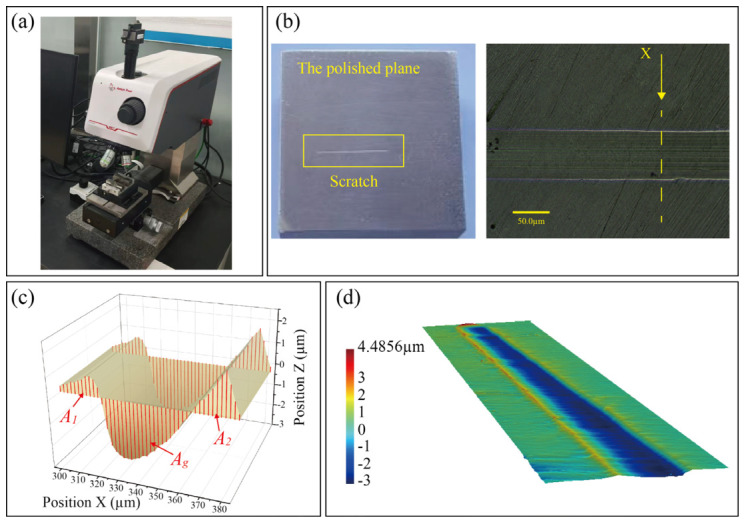
Scratch test experiment. (**a**) High−load scratch tester; (**b**) polished surface specimen and scratch morphology; (**c**) scratch cross−sectional profile; (**d**) scratch surface morphology.

To determine the furrow ratio coefficient *f*_ab_ under the present experimental conditions, single-particle scratch tests were performed under controlled conditions. Scratches were generated on a polished AlSi10Mg surface under the same normal load as that used in the force measurement experiments. The 3D morphology and cross-sectional profiles of the scratches were obtained using a laser confocal microscope, as shown in Figure 11.

From the cross-sectional profile analysis, the theoretical groove area *A_g_* and the plastic pile-up areas on both sides (*A*_1_ and *A*_2_) were extracted, and the value of *f*_ab_ was determined from Equation (27).

And the single-particle scratch test was intended to approximate the local abrasive-material interaction behavior rather than fully reproduce the collective dynamics of the magnetic abrasive brush. In practical MAF processes, abrasive particles are constrained by flexible magnetic chains and dynamic magnetic interactions, resulting in compliant multi-particle contact behavior. Therefore, the scratch test provides a simplified characterization of the local micro-cutting and plowing behavior under controlled contact conditions. In the present study, an averaged furrow ratio coefficient was adopted as a simplified approximation to characterize the overall material removal tendency during the polishing cycle. Although this assumption improves model tractability, it may introduce deviations during the early-stage roughness reduction process. The polished-surface scratch test still provides valuable comparative information regarding local abrasive-material interaction behavior and offers a controllable basis for estimating the relative contribution of plowing-induced deformation during magnetic abrasive finishing.

To ensure experimental repeatability, the magnetic abrasives were replaced every 15 min during the polishing process. The samples were periodically weighed every 30 min using an electronic balance with a resolution of 0.1 mg to determine the material removal mass. The experiments were performed on SLM-fabricated AlSi10Mg cylindrical internal channels using composite magnetic abrasives under a coil excitation frequency of 5 Hz. To ensure experimental repeatability and statistical reliability, each polishing experiment was repeated three times under identical conditions. The reported surface roughness and material removal results represent the average values of the repeated measurements, and the corresponding standard deviations are presented as error bars in the figures.

**Figure 12:**
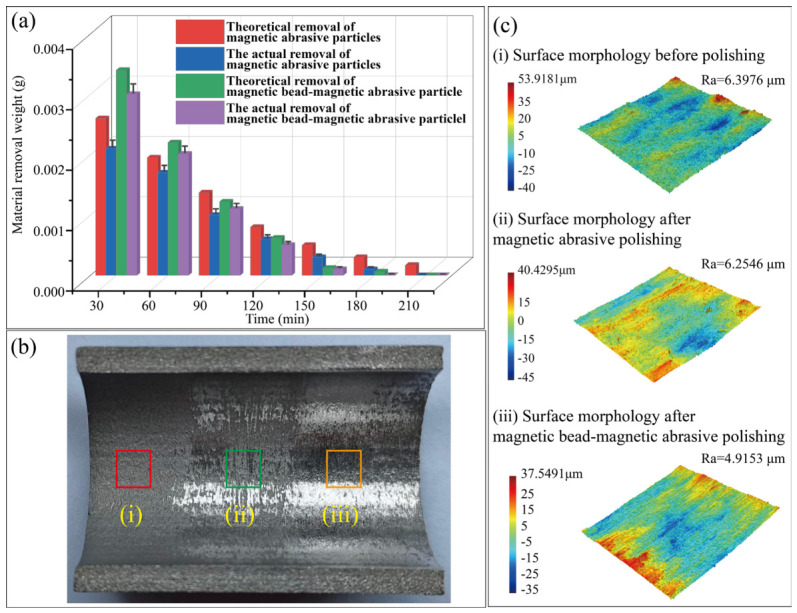
Experimental Results. (**a**) Material removal comparison of two polishing tools in an additively manufactured AlSi10Mg tube with an inner flow channel; (**b**) Polishing positions within the internal flow channel; (**c**) Surface topography.

As shown in Figure 12a, the material removal performance of MB–MAPs is consistently superior to that of MAPs at the early stage of polishing. At 30 min, the experimental material removal of MAPs is 2.1 × 10^−3^ g, whereas that of MB–MAPs reaches 3.0 × 10^−3^ g, corresponding to an improvement of approximately 42.9%. At 60 min, the improvement remains significant at 18.2%, while at 90 min, the enhancement decreases to about 10%. However, as the polishing process progresses, the advantage of MB–MAPs gradually diminishes. At 120 min, the material removal of MB–MAPs becomes slightly lower than that of MAPs, indicating that the process enters a fine finishing stage where the removal mechanism is dominated by surface smoothing rather than aggressive cutting.

To evaluate the predictive capability of the theoretical model, the relative error between theoretical and experimental values was calculated. For MAPs, the relative error ranges from 14.7% to 37.0% in the initial stage and increases significantly at later stages due to the extremely low material removal. In contrast, the error for MB–MAPs is generally lower, remaining within 10–25% over most of the polishing duration. These results indicate that the proposed model exhibits better predictive accuracy for the MB–MAP system, suggesting that the material removal mechanism under the composite magnetic field is more stable and consistent with theoretical assumptions.

## 4. Discussion

The results demonstrate that the MB–MAPs system achieves significantly higher material removal than conventional MAPs at the early stage, with an improvement of up to about 43% at 30 min. This enhancement supports the working hypothesis that magnetic balls reinforce the abrasive brush by increasing normal force and stabilizing abrasive motion, thereby promoting more effective micro-cutting. Similar effects have been reported in magnetic field-assisted finishing, where stronger magnetic chains lead to higher removal efficiency.

As polishing progresses, the advantage of MB–MAPs gradually diminishes. This trend indicates a transition in the dominant removal mechanism from micro-cutting to plastic deformation and surface smoothing, where the contribution of force enhancement becomes less significant. Such stage-dependent behavior is consistent with previous studies on abrasive finishing processes.

The theoretical model shows good agreement with experimental results, particularly for the MB–MAP system, suggesting that it captures the essential removal characteristics under composite magnetic fields. The increased deviation at later stages is mainly attributed to abrasive wear, elastic recovery, and measurement limitations when the removal approaches the resolution of the balance.

Overall, the findings highlight the effectiveness of MB–MAPs in improving early-stage polishing efficiency and provide guidance for process optimization. Future work should focus on incorporating abrasive evolution and adaptive control strategies to further enhance model accuracy and finishing performance.

Although the proposed model provides a mechanistic interpretation of the rolling-sliding transition behavior during magnetic abrasive finishing, several limitations remain. The present study adopts homogenized material properties and simplified statistical contact assumptions, while the actual SLM surface exhibits heterogeneous microstructures and dynamically evolving roughness topographies. Future work will focus on incorporating elastoplastic asperity evolution models, adhesive contact behavior, and time-dependent roughness evolution into the polishing framework. In addition, coupling between magnetic brush flexibility and multi-particle stochastic interactions will be further investigated to improve predictive capability for complex AM internal channels. To complement the mechanistic modeling and provide a more comprehensive assessment of surface integrity, Abbott–Firestone bearing area curve analysis [27] and additional surface functional parameters will also be employed to characterize the evolution of load-bearing characteristics and surface morphology following composite magnetic field polishing.

## 5. Conclusions

This work systematically investigates the surface finishing of AlSi10Mg internal channels fabricated by additive manufacturing using magnetic abrasive finishing under a composite magnetic field. The main conclusions are as follows:

(1) A mechanistic material removal model based on micro-cutting and plastic deformation is established and validated. Experimental comparison between MAPs and MB–MAPs shows good agreement, with relative errors of MB–MAPs generally within 10–25%.
(2) MB–MAPs significantly enhance early-stage material removal efficiency, improving by about 42.9% at 30 s and 18.2% at 60 s. The enhancement decreases with time, while surface quality is improved through more effective removal of asperities and adhered particles.
(3) The composite magnetic field ensures stable abrasive motion and full coverage in complex channels. However, the removal rate decreases from 3.0 × 10^−3^ g to below 1.0 × 10^−4^ g as polishing progresses, indicating a transition from dominant cutting to surface smoothing.
(4) The combined model and process provide a unified framework for understanding material removal behavior. Deviations at later stages are mainly due to abrasive wear, elastic recovery, and measurement limitations, offering guidance for process optimization of additively manufactured components.
(5) Since surface roughness and residual surface defects strongly influence the corrosion behavior of additively manufactured aluminum alloys, future work will further investigate the influence of composite magnetic field polishing on corrosion resistance and long-term surface stability in complex internal flow channels.

## Figures and Tables

**Table 1:** Internal flow channel properties of AlSi10Mg alloy prepared by SLM.

Performance Category	Specific Indicators	AlSi10Mg Typical Values/Characteristics
Microstructure	α-Al	Fine cellular/Columnar dendrites
	Si	A fine, continuous network structure is distributed along grain boundaries
Mechanical Properties	Elastic modulus	72 MPa
	Poisson ratio	0.33
	Yield strength	245 MPa
	Hardness	60–120 HB
	Mass density	2.68 g/cm^3^
	Tensile strength	330 MPa
	Elongation at break	6%
	Melting point temperature	700 °C
	Operating Temperature	350 °C

## Data Availability

The original contributions presented in this study are included in the article. Further inquiries can be directed to the corresponding author.

## References

[1] Özel T. (2025). Deep Learning-Based Applications in Metal Additive Manufacturing Processes: Challenges and Opportunities–A Review. Int. J. Lightweight Mater. Manuf..

[2] González-Henríquez C.M., Sarabia-Vallejos M.A., Rodriguez-Hernandez J. (2019). Polymers for Additive Manufacturing and 4D-Printing: Materials, Methodologies, and Biomedical Applications. Prog. Polym. Sci..

[3] Kladovasilakis N., Tsongas K., Tzetzis D. (2020). Finite Element Analysis of Orthopedic Hip Implant with Functionally Graded Bioinspired Lattice Structures. Biomimetics.

[4] Gebisa A.W., Lemu H.G. A Case Study on Topology Optimized Design for Additive Manufacturing. Proceedings of the First Conference of Computational Methods in Offshore Technology: COTech2017.

[5] Yap C.Y., Chua C.K., Dong Z.L., Liu Z.H., Sing S.L. (2015). Review of Selective Laser Melting: Materials and Applications. Appl. Phys. Rev..

[6] Blakey-Milner B., Gradl P., Snedden G., Brooks M., Pitot J., Lopez E., Leary M., Berto F., Du Plessis A. (2021). Metal Additive Manufacturing in Aerospace: A Review. Mater. Des..

[7] Zhang B., Li Y., Bai Q. (2017). Defect Formation Mechanisms in Selective Laser Melting: A Review. Chin. J. Mech. Eng..

[8] Anand M., Das A.K. (2021). Issues in Fabrication of 3D Components through DMLS Technique: A Review. Opt. Laser Technol..

[9] Sirwal S.A., Singh A.K. (2019). Analysis of the Surface Roughness for Novel Magnetorheological Finishing of a Typical Blind Hole Workpiece. J. Mech. Eng. Sci..

[10] Singh R.K., Singh D.K., Gangwar S. (2018). Advances in Magnetic Abrasive Finishing for Futuristic Requirements-a Review. Mater. Today Proc..

[11] Chang G.-W., Yan B.-H., Hsu R.-T. (2002). Study on Cylindrical Magnetic Abrasive Finishing Using Unbonded Magnetic Abrasives. Int. J. Mach. Tools Manuf..

[12] Zhang G., Zhao Y., Zhao D., Yin F., Zhao Z. (2011). Preparation of White Alumina Spherical Composite Magnetic Abrasive by Gas Atomization and Rapid Solidification. Scr. Mater..

[13] Zhang J., Sun Y., Su G., Li B., Du J., Zhang P., Xia Y. (2025). Magnetic Polishing and Grinding of Sintered Mn-Doped SiC/Al2O3/Fe Composite Particles. Ceram. Int..

[14] Archard J. (1953). Contact and Rubbing of Flat Surfaces. J. Appl. Phys..

[15] Archard J.F., Hirst W. (1956). The Wear of Metals under Unlubricated Conditions. Proc. R. Soc. A.

[16] Rabinowicz E., Dunn L.A., Russell P.G. (1961). A Study of Abrasive Wear under Three-Body Conditions. Wear.

[17] Garrison W.M., Garriga R.A. (1983). Ductility and the Abrasive Wear of an Ultrahigh Strength Steel. Wear.

[18] Zum Gahr K.H., Mewes D. (1983). Severity of Material Removal in Abrasive Wear of Ductile Metals. Proceedings of the International Conference on Wear of Materials.

[19] Zum Gahr K.-H. (1981). Formation of Wear Debris by the Abrasion of Ductile Metals. Wear.

[20] Guo S., Wang G., Yin Z., Li H., Zhang J., Sun Y. (2025). A Precision Polishing Method for 3D-Printed Complex Internal Flow Channel Components with Composite Magnetic Field Control. J. Manuf. Process..

[21] Wang L., Sun Y., Xiao Z., Yang F., Kang S., Liu Y., Zuo D. (2024). A Review of Magnetic Abrasive Finishing for the Internal Surfaces of Metal Additive Manufactured Parts. J. Manuf. Mater. Process..

[22] Medibew T.M., Agebo S.W., Deja M. (2025). Recent Research Progress in the Abrasive Machining and Finishing of Additively Manufactured Metal Parts. Materials.

[23] Ahmad S., Tian Y., Arora K. (2025). Magnetic Abrasive Finishing: Innovations and Possibilities. J. Manuf. Process..

[24] Johnson K.L. (1985). Contact Mechanics.

[25] Fang L., Kong X.L., Su J.Y., Zhou Q.D. (1993). Movement Patterns of Abrasive Particles in Three-Body Abrasion. Wear.

[26] Greenwood J.A., Williamson J.B.P.P. (1966). Contact of Nominally Flat Surfaces. Proc. R. Soc. A.

[27] Szwajka K., Trzepieciński T., Szewczyk M., Zielińska-Szwajka J., Barlak M. (2025). Investigating Resulting Surface Topography and Residual Stresses in Bending DC01 Sheet under Tension Friction Test. Lubricants.

